# Consistency in diagnostic suggestions does not influence the tendency to accept them

**Published:** 2012-09-30

**Authors:** Kees van den Berge, Silvia Mamede, Tamara van Gog, Jan van Saase, Remy Rikers

**Affiliations:** 1 Department of Internal Medicine, Erasmus Medical Centre, Rotterdam, the Netherlands; 2 Department of Psychology, Erasmus University Rotterdam, Rotterdam, the Netherlands

## Abstract

**Background:**

Studies suggest that residents tend to accept diagnostic suggestions, which could lead to diagnostic errors if the suggestion is incorrect. Those studies did not take into account that physicians in clinical practice will mainly encounter correct suggestions. The present study investigated residents’ diagnostic performance if they would first encounter a number of correct suggestions followed by a number of incorrect suggestions, and vice versa. It was hypothesized that more incorrect suggestions would be accepted if participants had first evaluated a series of correct suggestions.

**Method:**

Residents (*n* = 38) evaluated suggested diagnoses on eight written clinical cases. Half of the participants first evaluated four correct suggestions and then evaluated four incorrect suggestions (C/I condition). The other half started with the four incorrect suggestions followed by the correct suggestions (I/C condition).

**Results:**

Our findings show that the evaluation score in the C/I condition (*M* = 2.87, *MSE* = 0.14) equaled that in the I/C condition (*M* = 2.66, *MSE* = 0.14), *F*(1,36) = 1.09, *p* = 0.30, *ns*, meaning that consistency in preceding suggested diagnoses did not influence the tendency to accept subsequent diagnostic suggestions. There was, however, a significant interaction effect between case order and phase, *F*(1,36) = 11.82, *p* = 0.001, *η_p_^2^* = 0.25, demonstrating that the score on cases with correct suggestions was higher than the score on cases with incorrect suggestions.

**Conclusion:**

These findings indicate that consistency in preceding correct or incorrect diagnostic suggestions did not influence the tendency to accept or reject subsequent suggestions. However, overall residents still showed a tendency to accept diagnostic suggestions, which may lead to diagnostic errors if the suggestion is incorrect.

## Introduction

Several studies suggested that physicians tend to accept diagnostic suggestions for clinical cases.^1^–^3^ Such an inclination towards confirmation might, if the suggestion is incorrect, lead to diagnostic errors. Those studies, however, did not take into account that in everyday clinical practice, physicians are likely to encounter many correct suggestions before they are confronted with an incorrect suggestion. This study investigates whether the inclination towards accepting diagnostic suggestions is influenced by consistency in correctness of preceding diagnostic suggestions.

The publication of the Institute of Medicine report led to an increase in research on medical errors, and stimulated discussion on patient safety issues.^4^–^6^ The report showed that medical mistakes, the majority of which are related to treatment, cause many preventable deaths in the United States. Besides treatment-errors, diagnostic mistakes account for a substantial portion of medical errors. The rate of such diagnostic errors lies within the 10–15% range,^7^,^8^ and the clinical specialties of internal medicine and emergency medicine are believed to be most affected by them.^9^–^11^

Diagnostic errors have many causes but a substantial number of mistakes seem to stem from faults in physicians’ cognitive processes.^12^ These so-called ‘cognitive diagnostic errors’ may occur due to insufficient knowledge, but other factors, such as faulty gathering or interpretation of clinical data, and flawed verification of diagnostic hypotheses, have been pointed out as the main culprits.^12^ Several authors have discussed the potential of cognitive factors to cause diagnostic errors,^13^–^15^ and observational studies suggest that clinicians’ thinking errors may actually be involved in the majority of missed or delayed diagnoses.^12^,^16^

The discussion about the causes of cognitive diagnostic errors is ongoing,^17^ and may benefit from medical expertise research. This research suggests that diagnostic reasoning may be vulnerable to bias.^18^–^20^ For example, it has been demonstrated that physicians generate hypotheses in the beginning of patient contact, mainly through pattern recognition: similarities between the current and previously seen patients quickly bring one or a couple of diagnostic hypotheses to the physician’s mind, which are used to guide the search for additional evidence. This mainly automatic, non-analytical mode of reasoning occurs relatively effortless, and is the chief mode of reasoning when clinicians deal with routine problems.^20^ It is usually effective but, as it occurs largely without conscious control, generation of hypotheses based on pattern recognition may be influenced by multiple factors that remain unnoticed, making physicians more prone to bias and, consequently, to errors.^14^,^15^

Admitted patients often come with the diagnostic considerations of another medical professional (e.g., the general practitioner, a nurse, or the ambulance personnel). If physicians would tend towards accepting such suggestions, correct suggestions could facilitate fast and accurate diagnosis. However, even though such suggestions will often be correct, they may sometimes be wrong, and in that case, accepting diagnostic suggestions may lead to errors.^21^,^22^ A recent study showed that physicians indeed tend to accept diagnostic suggestions for written clinical cases.^1^ In that study, residents in internal medicine evaluated diagnostic suggestions for subsequently presented case-descriptions, which were all based on real patients and had a verified diagnosis. Half of the diagnostic suggestions were correct, and half of them were incorrect. Results showed that participants found it harder to reject an incorrect suggested diagnosis than to accept a correct suggested diagnosis. However, in that study, the correct and incorrect suggestions alternated, which is unlikely to happen in everyday clinical practice. That is, in clinical practice, the correctness of diagnostic suggestions is unlikely to alternate that often.

Based on research in medical expertise, it can be assumed that perceiving a consistent series of diagnostic suggestions might influence diagnostic decision making on subsequent cases. For instance, seeing a consistent series of correct diagnostic suggestions might lead to the expectancy that a next suggestion is also likely to be correct, and hence increases the chances that it is accepted even when incorrect.^23^–^25^ On the other hand, it is known that when physicians encounter inconsistencies or complexity in cases, they may return to a more deliberate mode of diagnostic reasoning.^23^,^25^ Accordingly, it can be hypothesized that, when inconsistencies between the suggested diagnosis and the findings in a case are noticed, this is likely to evoke a more critical approach towards such suggestions.

In the present study, it is hypothesized that more incorrect suggestions would be accepted if participants have first evaluated a number of correct suggestions than when no correct suggestions were evaluated prior to evaluating incorrect suggestions. Conversely, it is hypothesized that, if participants had first evaluated incorrect suggestions, they would become more critical about the suggestions, and hence will become more inclined to reject correct suggestions than when no incorrect suggestions were evaluated prior to evaluating correct suggestions.

## Method

### Participants

Thirty-eight internal medicine residents (mean age = 30.00, *SD* = 3.06 years; 23 women) from a university hospital in the Netherlands voluntarily participated in this study. The ethics review committee of the Department of Psychology, Erasmus University Rotterdam, approved this study. Participants were debriefed after the study.

### Materials

A set of eight written clinical cases, which were based on real patients and had confirmed diagnoses, was used in the study (see Appendix for an example). They were designed and validated independently by two experts in internal medicine and had been previously used in studies with internal medicine residents.^1^,^26^ The cases were presented to the participants in a booklet, showing one case per page. Each case description was preceded by a diagnostic suggestion. Immediately after reading each case description, the participants evaluated the diagnostic suggestion by indicating whether they agreed or disagreed.

The cases consisted of two series of four. One series with four correct suggested diagnoses and the other four with incorrect suggested diagnoses. Within each series, the cases were presented in a fixed order. There were two versions of the booklet; the cases were the same in both versions but the two series of cases were presented in a different order: half of the participants evaluated four correct suggested diagnoses, followed by four incorrect suggested diagnoses (C/I condition). The other half first evaluated the four incorrect suggested diagnoses, and then evaluated the four correct suggestions (I/C condition, see Table 1 for an overview of the materials).

After evaluating the suggested diagnoses, participants evaluated their experience with the diagnoses that were presented as suggested diagnoses on a seven point Likert-scale, ranging from (1) “no experience with the disease”, to (7) “highly experienced with the disease”. A short demographic questionnaire concerning gender and age completed the materials.

In order to ensure that cases with a correct suggestion did not differ in complexity from cases with an incorrect suggestion, a pilot study was conducted using the same cases. In this pilot, the cases were randomly presented to 15 participants in a booklet, showing one case per page. The participants were asked to read the case quickly but carefully and write their diagnosis, immediately after each case text. They were allowed 75 seconds to diagnose each case. For each correct diagnosis, a score of 1 point was assigned. When the diagnosis was incorrect, no points were given. Results showed the diagnostic performance on cases that were presented with correct suggestions in the main study (*M* = 2.80, *SD* = 0.77) did not significantly differ from diagnostic performance on cases that were accompanied by an incorrect suggestion (*M* = 3.27, *SD* = 1.03) in the main study, *t*(14) = 1.39, *p* > 0.05.

### Procedure

The study was conducted during a bimonthly educational session, which is part of the internal medicine residency training program in the Netherlands. These educational sessions that last one day, consist of lectures and discussions on a range of topics in internal medicine. Participation is voluntary and involvement of the attending residents is generally 100%. The instruction for evaluation of the cases was provided in the booklet: “Read the following cases quickly but carefully and indicate whether you agree or disagree with the diagnosis”. Based on a previous study, participants were given 75 seconds to evaluate each diagnosis.^1^ Time was kept by an experiment-leader who told the participants to continue to the next case after every 75 seconds. The whole procedure took about 20 minutes.

### Data analysis

Mean experience ratings with the four correct and the four incorrect suggested diagnoses were calculated, resulting in experience scores ranging from 1 (i.e., no experience) to 7 (i.e., high experience) for both correct and incorrect suggested diagnoses. An independent samples *t*-test was used to compare experience with the diseases presented as (in)correct diagnostic suggestions between conditions. A paired samples *t*-test was used to compare experience with the diseases presented as correct or incorrect diagnostic suggestions within conditions. Participants’ data on diagnostic decisions were scored as follows: for each correct evaluation (i.e., agreeing with the correct suggested diagnosis, disagreeing with the incorrect suggested diagnosis) a score of 1 point was obtained. So, a maximum score of 8 points could be obtained: 4 points for rejecting incorrect diagnoses and 4 points for accepting correct diagnoses. Data on diagnostic decisions were submitted to a mixed-design 2 × 2 analysis of variance (ANOVA) with case order (i.e., C/I or I/C) as a between-subjects factor and the phase of the experiment (i.e., the first four cases compared with the last four cases) as a repeated measure.

For all analyses, a significance level of 0.05 is used. For the ANOVA, *η_p_^2^* is reported as a measure of effect size with values of 0.01, 0.06, and 0.14, corresponding to small, medium, and large effect sizes respectively. For *t*-tests, *d* is reported as a measure of effect size with values of 0.20, 0.50, and 0.80, corresponding to small, medium, and large effect sizes, respectively.^27^

## Results

### Participants’ characteristics and experience with suggested diagnoses

Participants’ characteristics were similar between conditions, as shown in Table 2. Experience (range: 1–7) with diagnoses presented as incorrect diagnostic suggestions in the C/I condition equaled that of experience in the I/C condition. Experience with the diagnoses presented as correct suggestions was also similar between conditions. Within both conditions, experience with diagnoses presented as incorrect suggestions (C/I: *M* = 3.90, *SD* = 0.96; I/C: *M* = 3.79, *SD* = 0.74) exceeded experience with the diagnoses presented as correct suggested diagnoses (C/I: *M* = 3.04, *SD* = 0.96; I/C: *M* = 3.07, *SD* = 0.74) with, *t*(18) = 4.43, *p* < 0.05, *d* = 0.92, in the C/I condition, and *t*(18) = 4.43, *p* < 0.05, *d* = 0.96 in the I/C condition.

### Diagnostic scores

There was no significant main effect of case order: the diagnostic evaluation score in the C/I condition (*M* = 2.87, *MSE* = 0.14) equaled that score in the I/C condition (*M* = 2.66, *MSE* = 0.14), *F*(1.36) = 1.09, *p* = 0.30. There was also no main effect of phase: evaluation score on the first 4 cases (*M* = 2.68, *MSE* = 0.14) equaled the score on the last 4 cases (*M* = 2.84, *MSE* = 0.17), *F*(1,36) < 1, meaning that consistency in preceding suggested diagnoses did not influence the tendency to accept subsequent diagnostic suggestions.

**Figure 1:**
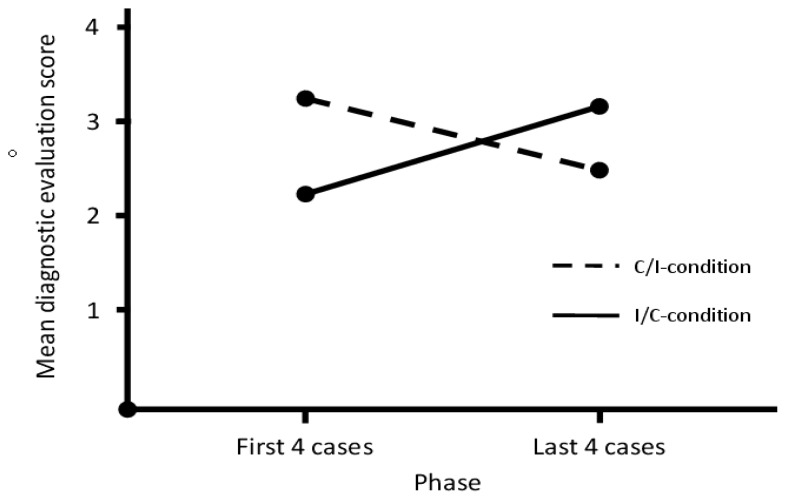
Interaction between condition and phase of case-presentation.

There was, however, a significant interaction effect between case order and phase (see Figure 1), demonstrating that within both conditions the score on cases with correct suggestions (C/I: *M* = 3.21, *MSE* = 0.20, I/C: *M* = 3.16, *MSE* = 0.25) was higher than the score on cases with incorrect suggestions (C/I: *M* = 2.53, *MSE* = 0.25, I/C: *M* = 2.16, *MSE* = 0.20), *F*(1,36) = 11.82, *p* = 0.001, *η_p_^2^* = 0.25.

## Discussion

The present study investigated whether residents’ tendency to accept diagnostic suggestions on written clinical cases could be influenced by a more consistent presentation of the suggestions.^1^ It was hypothesized that, if a series of diagnostic suggestions would prove consistent with subsequently read case-descriptions (i.e., the suggestions were correct) this would lead to an increased confidence in the source of the suggestions, resulting in increased accepting of subsequently presented incorrect suggestions.^24^ This tendency would lead to diagnostic mistakes, revealed by a lower diagnostic evaluation score on cases with incorrect suggestions than that of participants who were not first exposed to correct suggestions. Conversely, it was hypothesized that if participants would experience inconsistencies between a diagnostic suggestion and subsequent case findings, this would lead to a more critical appraisal of subsequently presented cases with correct suggestions,^23^,^25^ resulting in a lower score on these cases by participants who had first seen incorrect suggestions than participants who were not first exposed to incorrect suggestions.

In contrast to these hypotheses, participants did not accept more incorrect suggestions after encountering a series of correct suggestions than when no prior correct suggestions had been encountered. Likewise, when participants had first evaluated incorrect suggestions they did not make more mistakes on subsequent cases with correct suggestions than their colleagues who first saw those cases with correct suggestions. Therefore, consistency in diagnostic suggestions does not seem to contribute to diagnostic errors in later cases. The significant interaction effect between condition and phase, however, showed that the rate of accepted incorrect diagnoses, although equal between conditions, was substantial within both conditions. That is, 52% of incorrect diagnoses and 80% of correct suggestions were accepted. This tendency to accept diagnostic suggestions may lead to diagnostic errors if the suggestion happens to be incorrect.^1^,^14^,^15^

It could be argued that the tendency to accept incorrect suggestions in this study results from differences in case complexity, because different cases accompanied correct and incorrect diagnostic suggestions. However, a pilot study among similar participants revealed no differences in diagnostic performance on those cases, indicating differences in case complexity are unlikely to explain this finding. In addition, differences in experience with the diagnoses presented as correct and incorrect diagnoses can also not account for participants’ greater difficulty with rejecting incorrect suggestions. That is, participants experience with diagnoses presented as incorrect suggestions even exceeded experience with diagnoses presented as correct suggestions. Therefore, participants are potentially able to reject these incorrect diagnostic suggestions. This implies that the tendency to accept diagnostic suggestions indeed might be hard to resist.^1^

The question is why consistency in preceding diagnostic suggestions did not influence diagnostic decisions on subsequently presented suggestions, is not easy to answer. A potential explanation might be that exposing participants to only four cases to build up confidence or distrust, might have been insufficient. However, studies on routine behaviour have shown that engaging in as few as two repetitive tasks could be enough to persuade naïve participants to “stick to the routine”.^28^ In addition, since the diagnostic decision score on cases with correct suggestions was not perfect (i.e., approximately 80%), it could be argued that participants were not as confident in their case evaluations as was anticipated. However, scores of about 80% on cases with correct suggested diagnoses are consistent with findings in previous studies,^1^–^3^. Still, the cases that were used were not simple, which may explain the score on cases with correct diagnostic suggestions. Perhaps the use of very uncomplicated cases would have increased the score on cases with correct suggestions, possibly resulting in higher confidence in the suggestions.

Future studies could attempt to directly measure participants’ confidence in their diagnostic conclusions. Although several experimental studies have addressed physicians’ confidence, 2,3,29 direct insights in physicians’ confidence in their diagnostic conclusions on cases with suggested diagnoses and the actual accuracy of their diagnoses has, to the best of our knowledge, not been experimentally investigated, and might lead to further improvement of our understanding of the handling of diagnostic suggestions.

The present study has important implications for clinical practice and medical education. Consistency in diagnostic suggestions did not influence the acceptance of subsequently presented diagnostic suggestions. However, in both conditions, a substantial number of incorrect suggestions were accepted. Still, in practice diagnostic suggestions are probably correct most of the time; ignoring them would be ineffective and even unwarranted. It would therefore be much better to train physicians to identify those situations in which a diagnostic suggestion might be faulty. Research on the role of reflection in clinical practice can play an important role to help physicians to identify those situations.^26^

In conclusion, this study showed that physicians’ tendency to accept diagnostic suggestions is independent of the correctness of preceding suggestions. Since the inclination towards accepting suggestions can, if the suggestions are incorrect, lead to errors, further study of causal and protective mechanisms should be conducted.

### Key learning points

- Diagnostic errors offer a substantial contribution to medical mistakes.
- Faults in individual physicians’ cognitive processes, such as errors resulting from confirmation bias are considered an important cause of diagnostic error.
- Residents exhibit confirmatory tendencies since they tend to accept diagnostic suggestions.
- The accepting of incorrect diagnostic suggestions may lead to diagnostic errors.
- This tendency to accept diagnostic suggestions is not influenced by consistency in preceding diagnostic suggestions.

## Figures and Tables

**Table 1:** Suggested diagnoses and correct diagnoses for the cases used in the study

C/I condition		I/C condition	

Suggested diagnosis	Correct diagnosis	Suggested diagnosis	Correct diagnosis
Aortic dissection	Aortic dissection	Q fever	Viral infection
Inflammatory bowel disease	Inflammatory bowel disease	Legionnaire’s disease	Pneumococcal pneumonia
Neurosyphilis	Neurosyphilis	Ulcerative colitis flare-up	Clostridium colitis
Primary sclerosing cholangitis	Primary sclerosing cholangitis	Liver metastasis	Liver cirrhosis
Q fever	Viral infection	Aortic dissection	Aortic dissection
Legionnaire’s disease	Pneumococcal pneumonia	Inflammatory bowel disease	Inflammatory bowel disease
Ulcerative colitis flare-up	Clostridium colitis	Neurosyphilis	Neurosyphilis
Liver metastasis	Liver cirrhosis	Primary sclerosing cholangitis	Primary sclerosing cholangitis

**Table 2:** Characteristics of the participants

Characteristic	CI condition	IC condition	*p* value
Age (yrs)	*M* = 30.26, *SD* = 3.07	*M* = 29.74, *SD* = 3.11	0.60
Average experience correct suggestions	*M* = 3.04, *SD* = 0.96	*M* = 3.07, *SD* = 0.96	0.93
Average experience incorrect suggestions	*M* = 3.92, *SD* = 0.96	*M* = 3.79, *SD* = 0.77	0.64

## Appendix 1

Case of liver cirrhosis (correct diagnosis, to be confounded with liver metastasis (incorrect diagnosis).

History: A 45-year old lawyer complains about ongoing pain in the upper abdomen. The patient relates the pain to stress due to a decreasing number of clients and his divorce, now 2 years ago. He has sex with prostitutes on occasion but has been impotent lately. He smokes 40 cigarettes a day and drinks substantial amounts of alcohol. His medical history includes surgery for prostatic cancer 5 years ago, no food intolerances.

Physical examination: pale man, Blood Pressure: 110/69, Heart Rate: 85 beats per minute, Temperature: 37 ºC.

Thorax: heart and lungs: normal. Spider naevi present.

Abdomen: mild distension of the abdomen, percussion normal. Palpable liver with irregular surface. No splenomegaly.

Extremities: ankle edema

Testicles: very small

Laboratory testing: Hemoglobin 5.0 mmol/L (8,6–10,5); ESR 44 mm/h (<20); Sodium 138 mmol/L (135–145); Potassium 3.6 mmol/L (3,5–5,0); ALAT 120 U/L (<41); ASAT 84 U/L (<37); LDH 800 U/L (<450); y-GT 250 U/L (<50); Alkaline Phosphatase. 200 U/L (<120); Bilirubin 42.7μmol/L (<17).
